# Gait Event Detection on Level Ground and Incline Walking Using a Rate Gyroscope

**DOI:** 10.3390/s100605683

**Published:** 2010-06-04

**Authors:** Paola Catalfamo, Salim Ghoussayni, David Ewins

**Affiliations:** 1 Centre for Biomedical Engineering, Faculty of Engineering and Physical Sciences, University of Surrey, Guildford, Surrey, GU2 7XH, UK; E-Mails: s.ghoussayni@surrey.ac.uk (S.G.); d.ewins@surrey.ac.uk (D.E.); 2 School of Engineering, Universidad Nacional de Entre Ríos, Argentina

**Keywords:** gait event detection, Initial Contact, Foot Off, gyroscope

## Abstract

Gyroscopes have been proposed as sensors for ambulatory gait analysis and functional electrical stimulation systems. Accurate determination of the Initial Contact of the foot with the floor (IC) and the final contact or Foot Off (FO) on different terrains is important. This paper describes the evaluation of a gyroscope placed on the shank for determination of IC and FO in subjects walking outdoors on level ground, and up and down an incline. Performance was compared with a reference pressure measurement system. The mean difference between the gyroscope and the reference was less than −25 ms for IC and less than 75 ms for FO for all terrains. Detection success was over 98%. These results provide preliminary evidence supporting the use of the gyroscope for gait event detection on inclines as well as level walking.

## 1. Introduction

Conventionally, the gait cycle is divided into stance and swing phases. The Initial Contact (IC) and end of contact or Foot Off (FO) represent the start and end of these phases. Their detection is used in a variety of applications such as the analysis of spatio-temporal gait parameters and for the time normalization of gait data, for example, kinetic, kinematic or EMG. These events have also been proposed and evaluated for the control of functional electrical stimulation (FES) orthoses applied to correct drop foot [1,2].

Many detection algorithms have been proposed and evaluated using optoelectronic [3–6] or kinetic [7] equipment normally found in gait laboratories. However, for applications where long term, outdoor measurements are needed, such as ambulatory systems measuring activities of daily living, or for FES systems, it is important to use equipment that is cosmetic, portable, robust and ideally inexpensive. Sensors such as accelerometers and gyroscopes, which facilitate the measurement of kinematic variables, have been proposed for ambulatory gait analysis, for example: for measuring or estimating spatio-temporal gait parameters [8,9] and joint angle measurement [10–12]; and the reconstruction of the sagittal trajectory of limb segments [13]. These sensors provide continuous information during the gait cycle and they can be used for subjects walking barefoot. Moreover, recent technological developments have improved these sensors making them smaller, lighter, less expensive, and with relatively low current consumption, so appealing for long term, outdoor ambulatory applications.

Tilt sensors [14], accelerometers [15–17] and gyroscopes [18–20] have also been proposed for determination of gait events. Investigations into their performance continue as the number and type of sensors, and their positioning and detection algorithms, are evaluated for different applications.

Gyroscopes placed on the shank have been proposed: as part of ambulatory gait analysis systems [10,11,13,21]; as sensors for triggering [19] and feedback [12] of FES systems; as part of systems used to classify leg motion [22] and for a control system for lower limb prostheses and orthoses [23]. Locating the gyroscope on the shank offers some advantages over other locations, for example there is less soft tissue movement on the anterior aspect of the shank than on the thigh [24] and the signal is less variable between subjects for shank signal with respect to the foot signal [25]. The gyroscope may be worn under clothes, improving the system cosmesis and there is no need for footwear or footwear adaptations. For those applications that require detection of gait events, a detection system consisting of only one sensor has practical advantages in terms of cosmesis, cost, ease of placement, and time required to don and doff. Moreover, the gyroscope placed at the shank has proven to be acceptably accurate in healthy [18,19] and pathological gait for detection of gait events when walking on level ground [19,26,27]. However, the evaluation of detection using a gyroscope placed on the shank for subjects walking on different terrains is still pending.

Previous work has evaluated the detection of gait events for incline walking using one gyroscope placed on the foot [9,28]. However, as noted above, the location of the gyroscope on the shank presents some advantages in terms of ease of use and cosmesis over its location on the foot. Also, both studies reported the overall results considering ramp up and ramp down walking (and level ground for [9]) as one condition, hence the difference in detection between incline up, incline down and level ground was not assessed.

As there are major differences in the angle of the knee at IC and FO for walking up and down inclines with respect to level ground ambulation [29–31], it is possible that these changes may affect the detection of events using shank angular velocity. The purpose of this study was to evaluate the detection of IC and FO, using one gyroscope placed on the shank of subjects walking outdoors, on a path that included level ground and incline walking.

This study is part of a project at the University of Surrey directed at the evaluation of gyroscopes as sensors for outdoor gait event detection and their use for functional electrical stimulation drop foot correction systems. Previous research at the University included the evaluation of a gyroscope placed on the foot above the metatarsals in unimpaired subjects and in subjects with drop foot [32], and the evaluation of a gyroscope placed on the shank also in unimpaired subjects and subjects with drop foot when walking on level ground [19]. Both studies evaluated the detection in adults. This study extended the scope of application to children, with the evaluation covering both level ground and incline walking.

## 2. Method

### 2.1. Subjects

Seven subjects (five females and two males, 8–16 years of age, 1.38–1.87 m in height and 33.3–65.3 kg in mass) without discernable gait abnormalities participated in the study. The protocol was explained to the subjects and their parents and a consent form was signed by every parent and each subject. The Local Research Ethics Committee reviewed and approved this study.

### 2.2. Protocol

The subjects walked at a self selected ‘normal’ speed, wearing the shoes they use routinely for daily activities. The walking circuit started inside the gait laboratory, with subjects walking a short distance towards the door to the outside of the building. Once outside, they walked down an incline (7 m long, with an inclination of approximately 9°) and continued walking outside (on pavement) for approximately 150 m. After a short break, the subjects repeated the walking on pavement and then up the incline and into the laboratory. Only the data collected outside the laboratory was used for the analysis.

### 2.3. Gyroscope Data

A single axis gyroscope (ENC 03J, Murata Manufacturing Co., Ltd, Nagaokakyo-shi, Kyoto, Japan) was placed on the anterior aspect of the shank of the dominant leg, 5 to 10 cm below the tibia tuberosity, aligned with the long axis of the tibia and positioned so as to measure the angular velocity of the shank in the sagittal plane. A pressure measurement insole was inserted inside each shoe (F-Scan® Mobile system, Tekscan Inc., South Boston, M.A., USA). The subjects also carried a small rucksack, containing the conditioning box for the gyroscope sensor and two dataloggers. One datalogger was used for collection of gyroscope and synchronization data (AD128C, Omega Engineering Inc., Stamford, CT, USA) and the other for the pressure measurement system. The data sources were synchronized using an external switch and sampled at 125 Hz.

The gyroscope was mounted in a small box (32 × 22 × 17 mm), which was attached by Velcro to a strap wrapped around the shank. The output was conditioned in order to match the signal to the input range of the datalogger.

A digital filter (second order Butterworth low pass filter applied backwards and forwards, with a cut off frequency of 35 Hz) was applied offline to the collected angular velocity signal. Researchers have used a variety of cut off frequencies for shank angular velocity measured from a gyroscope. Tong and Granat [33], for example, used a low pass filter with frequency cut off of 4 Hz, while Mayagoitia *et al.* [13] used a cut off frequency of 3 Hz, and Nene *et al.* [34] used 5 Hz, although none of these researchers used the signal for event detection. When studying the spectrum of the signal, Ghoussayni [35], found a significant component at around 0.8 Hz and less significant harmonics up to 12 Hz, but did not use a filter in the final set up when detecting events. Henty [32] used a cut off frequency of 31 Hz when using the angular velocity of the foot for gait event detection. Since there were no clear guidelines on the cut off frequency to be used, its influence on the detection of gait events was investigated further.

Gyroscope data obtained from one unimpaired subject (female, 13 years of age) and one subject with cerebral palsy (female, 9 years of age, mild diplegia with left side more affected than right) were used. IC and FO were manually determined (by visually inspecting the signal) from the raw gyroscope data and from filtered gyroscope signals at 9 different cut off frequencies (40, 35, 30, 25, 20, 15, 12, 10 and 5 Hz). It was considered that frequencies below 5 Hz would limit the information carried in the signal especially at the time of the rapid loading events and that this would have an adverse effect on detection, so evaluation of frequencies lower than 5 Hz was not considered. A total of 34 IC events and 32 FO events were detected from the unimpaired subject data, while 15 IC and 13 FO events were detected from the data of the subject with cerebral palsy (CP). The absolute mean difference between the detection using raw data and each of the filtered data are shown in Tables 1 and 2 for IC and FO respectively.

It is necessary to emphasise that the signals were filtered with an effectively zero-phase-shift filter, so that the differences in detection are due to the magnitude change of the signal caused by filtering.

**Figure 1.**
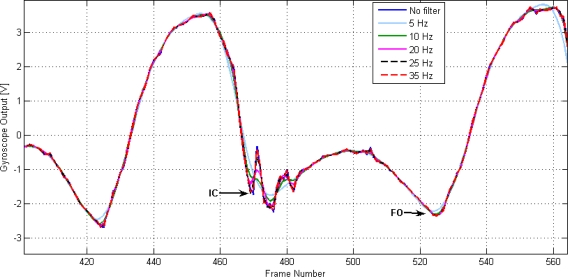
The effect of filtering using different cut off frequencies on the gyroscope signal from an unimpaired subject walking on level ground.

**Figure 2.**
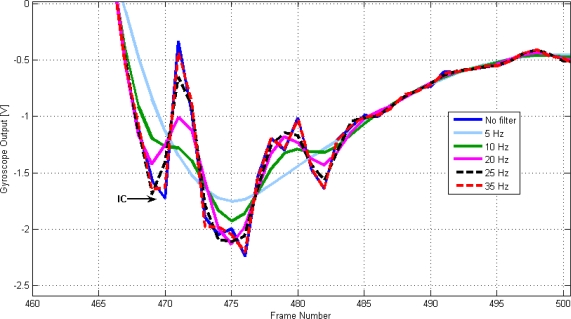
The effect of different cut off frequencies on the detection of IC. The data corresponds to an unimpaired subject walking on level ground.

Figures 1 and 2 demonstrate the effect of filtering on the detection of IC and FO using the gyroscope signal from one unimpaired subject.

The selection of the filter cut off frequency does require a compromise. A lower cut off frequency will provide a reduction in oscillations that will improve automatic detection; however, a higher cut off frequency will result in the less distortion due to filtering. From Tables 1 and 2, the event most affected by the filter is IC. In terms of IC, Table 1 suggests that cut off frequencies greater than 20 Hz produce minor changes in the overall delay. However, when reviewing all the data, a frequency of 20 Hz produced delays of up to 32 ms for some IC events, whereas a cut off frequency of 35 Hz gave a maximum error of 8 ms for all the steps analysed and for both subjects. This was the cut off frequency used, at the expense of adding extra rules in the algorithm to avoid false detections due to noise and high frequency signal components.

The specifications of the ENC 03J gyroscope states that the output at zero angular velocity changes with temperature and the use of a high pass filter to account for the drift is suggested. For this project, the null output of the circuit was measured before starting each walk and this value was used as the zero. Since each walk would not take longer than 5 minutes, the change in temperature during the walk was considered negligible. However, if the software was implemented into on-line measuring system to be used for extended periods of time, a high pass filter should be used to account for drift due to changes in temperature.

**Figure 3.**
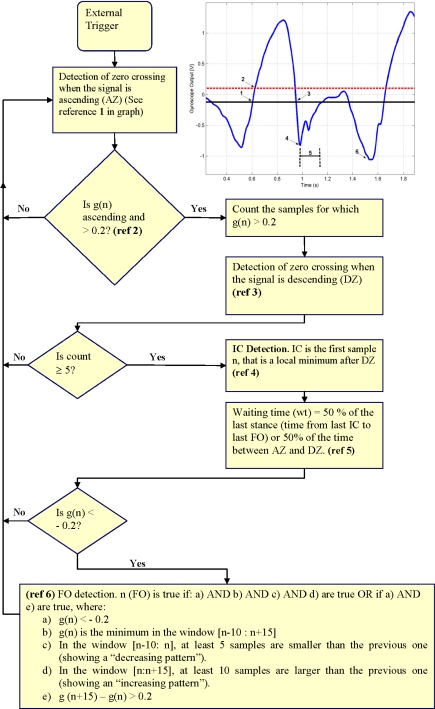
Flowchart of the algorithm used for detection of Initial Contact (IC) and Foot Off (FO) from the gyroscope signal. The graph corresponds to data collected from one subject who participated in the study. g(n) is the value of the gyroscope signal at the sample n; AZ: ascending zero crossing; DZ: descending zero crossing; and wt: waiting time. The numbers in the graph relate to steps of the algorithm: (1) detection of ascending zero crossing; (2) detection of signal exceeding threshold of 0.2 V; (3) detection of descending zero crossing; (4) IC detection; (5) waiting time; and (6) FO detection. After FO detection, the algorithm starts again.

A rule-based algorithm was written using Matlab® (Student Version 6.5, The Mathworks, Inc., Natick, MA, USA) for detection of events. Figure 3 shows a flowchart of the algorithm and a typical gyroscope signal from one of the subjects. The rules for the algorithm as well as the parameters chosen were determined empirically using preliminary data from two of the subjects who participated in this study.

The determination of IC and FO events is based on the detection of two negative peaks in the shank angular velocity signal. The algorithm evaluates each sample sequentially, starting from the onset of the external trigger. Initially, the algorithm searches for the swing phase of the cycle. When the gyroscope signal exceeds 0.2 V for at least 40 ms (5 samples for the sampling frequency used in this algorithm), the algorithm considers that swing phase has been detected (Reference 2 in Figure 3). The first negative minimum after swing is defined as IC (Reference 4 in Figure 3). Around the time of IC and briefly afterwards, the gyroscope signal may present further negative peaks related to events during the loading response. In order to avoid false detection of FO during that time, a “waiting time” was set during which no search for FO was carried out (Reference 5 in Figure 3). The waiting time was set to be 50% of the duration of the positive wave if this is the first step analysed or 50% of the last stance phase. Once this waiting time is over, every sample is evaluated as a possible FO. In this case, FO is defined as the sample that represents a minimum in a window of 200 ms (25 samples for the sampling frequency used in this algorithm), that is preceded by a decreasing (more negative voltage) trend in the signal and followed by an increasing (more positive voltage) trend, or it represents a minimum in the same window and it is followed by a rapid change towards positive values (indicating the extension of the knee), see reference 6 in Figure 3. Once FO has been determined the algorithm starts again, by looking for the following positive wave. The flowchart shown in Figure 3, is based on the sampling frequency of 125 Hz used in this study. If a different sampling frequency is selected, parameters would need to be adjusted accordingly.

### 2.4. Reference Systems

As a reference system, the FScan Mobile System was used. It consists of two insoles with a maximum of 960 different pressure sensing locations (sensels), conditioning boxes and a dedicated datalogger for data collection and storage. The insoles are light, thin and unobtrusive in the shoe. The total weight of the equipment is approximately 1.5 kg. The system has been shown to have acceptable accuracy for detection of IC and FO when using a Contact Area method [36].

The Contact Area method (described in detail in [36]) is based upon determining how much of the area of the insole has a non zero pressure reading at an instant in time. To detect the time when the loaded area starts to increase at Initial Contact, and the time when it decreases as the foot is lifted, an estimation of the area loaded when the foot was not in contact with the floor (area loaded during swing phase, ALSw) and of the area loaded during stance, ALSt, were calculated. Although nominally ALSw would be zero, during a trial it is possible that some areas of the insole become constantly loaded (for example, if the insoles move crinkles could appear near the edge). The estimated total loaded area is calculated as the difference between ALSt and ALSw. A threshold of 5% is then applied to the estimated total loaded area for detection of IC and FO. Hence, IC is determined as the first sample for which the total loaded area exceeds this threshold and FO is determined as the first sample, after stance, for which the total loaded area falls below this threshold.

### 2.5. Data Analysis

Once the events were determined for each method, the comparison was performed, separately for each type of terrain, by calculating the differences in time between the detection for each step analysed. The differences in timing between the Contact Area (CA) and the Gyroscope (GD) detection algorithms were calculated by subtracting the time of GD detection from that of CA detection. Also, in order to avoid misleading results due to cancellation of positive and negative values when averaging, the absolute mean difference (AMD) for each step was calculated. The differences and the AMD for all the steps for each subject were then averaged so that a single value was obtained for each subject and each condition. In order to compare the results with previously reported data, the mean differences for all the subjects were averaged and reported together with the 95% confidence interval (CI). The 95% CI was calculated as [37]:

$$\mathrm{CI}=X\pm t_{1-\alpha /2}\;s/\sqrt{n}$$

where: X is the estimated mean

s is the estimated standard deviation
n is the number of measures used to calculate the mean
t_1–__α/2_ is the “Student’s t”, its value depends on the probability level chosen and on the degrees of freedom upon which s is based [37].

In addition, the distributions of the differences were plotted in histogram form. For each event, the number of events *versus* the time difference expressed in ms (calculated in the range from −200 to 200 ms, divided in 10 ms interval) was calculated. Positive differences indicate that the GD method detected the event earlier than CA.

The differences were also analysed statistically. In order to test if the mean of the results for the three terrains (level walking, incline up and incline down) were statistically different, a Friedman test (level of significance p < 0.05) was applied. The Friedman test was selected since it is a nonparametric test that allows the comparison between three paired groups. It does not compare each pair of results, for example level walking and incline up walking, but the entire family of terrains. In order to analyse each pair, a Dunn post test was used. If the Friedman test found significant differences across the three groups, the Dunn post test determined which pair of terrains showed a significant difference. The analysis was performed using the GraphPad Instat® software (Version 3.05, GraphPad Software Inc., San Diego, CA, USA).

Finally, the success in detection was calculated. An error in detection was defined as an event missed or wrongly detected (as an extra event in a gait cycle for which another—correct—event had been detected). Then, the “success in detection” was calculated as the total number of events correctly detected by the gyroscope divided by the total events detected by the reference method and multiplied by 100.

## 3. Results and Discussion

The algorithm used in this study is rule-based and evaluates each sample sequentially, which facilitates the conversion into an on-line algorithm, if the application requires it. Also, it has been reported that a rule–based algorithm performed nine times faster than an algorithm based on wavelets analysis [27], which represents an advantage for on-line systems. The rules derived from data of two subjects were applied to the remaining five subjects.

Seven subjects completed the trial that included going up the incline and six completed the trial going down the incline. The total number of events analysed were 455 IC and 438 FO for level ground walking, 77 IC and 70 FO for walking up the incline, and 51 IC and 48 FO for walking down the incline.

Subjects walked at their self selected speed. The mean stride time for different terrains was (mean ± sd) 1.07 ± 0.09 s for incline down walking, 1.08 ± 0.10 s for level walking and 1.10 ± 0.10 s for incline up walking.

**Figure 4.**
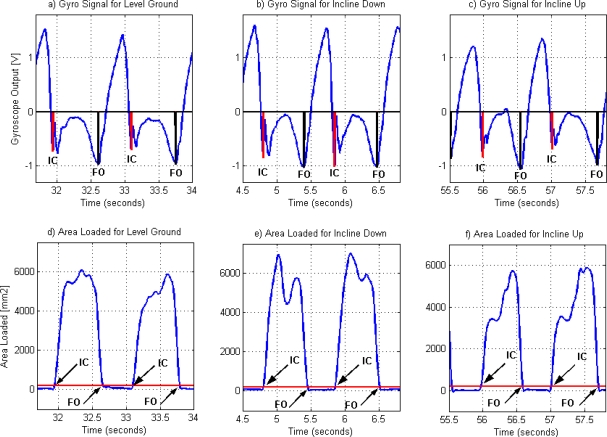
Gyroscope (a, b and c) and Contact Area loaded (d, e and f) signals from Subject 1. Vertical lines in the gyroscope graphs indicate the characteristic features of the gyroscope signal used for the detection of IC and FO events. (a) and (d) are the gyroscope and contact area loaded signals for the subject walking on level ground; (b) and (e) correspond to walking down the incline; and (c) and (f) correspond to walking up the incline.

Figure 4 shows a typical gyroscope and contact area signal for the three walking conditions, with the corresponding event detection. Although the signals for angular velocity present similar patterns, the angular velocity of the shank for walking up the incline (Figure 4c) shows differences with respect to the other two. In particular, the signal shows a zero crossing during the stance phase of gait, and the amplitude of the characteristic negative peaks following IC is smaller.

### 3.1. Mean Differences

The absolute mean difference, the mean difference and 95% confidence interval (CI) in detection (expressed in ms) for all the subjects and the three walking conditions are shown in Table 3. The p value from the Friedman test for IC was 0.052, considered not significant. The p value for the Dunn post test for the comparison of each pair of terrains was in all cases greater than 0.05, again considered not significant. In the analysis for FO, the Friedmann test for the three walking conditions gave a p value of 0.002, and the results of the post test revealed that the difference between the incline up and the incline down conditions was significant (p < 0.01), while the comparisons between the two incline conditions and level walking were not significant (p > 0.05).

The range of results for this study for the level ground condition are comparable to those found by other investigators who used a gyroscope, even when the reference methods used varies across studies (indoor evaluations make use of force platforms or optical systems while the present study used the F-Scan system for outdoor evaluation). Ghoussayni *et al.* [28] evaluated the event detection by a gyroscope placed on the foot of five unimpaired adults walking on level ground using a rule base algorithm and compared it with detection from optical systems. The mean difference between the methods was −7 ms for IC and 56 ms for FO. Aminian *et al*. [26] studied the signal from a gyroscope placed on the shank and used wavelet analysis [18] for detection of gait events. The detection was performed in eleven healthy subjects and compared with data from force platforms and optical systems. The mean difference and standard deviation for detection of IC was −16.6 ± 11.9 ms and for FO it was 3.7 ± 26.5 ms. Also, the present results are in the order of others obtained for different sensors evaluated. Selles *et al.* [16], for example, evaluated the detection of events using two uni-axial accelerometers placed on the shank of 15 healthy adults and compared it with detection using force platforms. They found a mean difference and standard deviation for IC of 34 ± 16 ms (with a CI of [3; 66]) and for FO of 19 ± 27 (with a CI of [−36; 76]). Smith *et al.* [38] evaluated force sensing resistors for detection of gait events in children. The detection was performed in four children with cerebral palsy (ages ranging from 7 to 13) and compared with detection from an optical system. The results showed a mean difference with respect to the optical system of −30 ± 125 ms for IC and 35 ± 80 ms for FO.

Two investigations were found in the literature that evaluated the detection of events by a gyroscope (the gyroscope was placed on the foot in both cases) in subjects walking on ramps (inclines).

Ghoussayni *et al.* [28] evaluated the detection of events by a gyroscope placed on the anterior side of the foot of five healthy adults walking up and down a ramp (inclination of 7°), comparing detection from an optical system. The overall mean difference was −11 ms for IC and 69 ms for FO. Sabatini *et al.* [9] used a gyroscope placed on the instep of the foot of two healthy adults walking over a treadmill. Five inclines, including level walking, were used (−5%, 0%, 5%, 10% and 15%). The gyroscope detection was compared to force sensing resistors placed under the heel and the first toe. The overall mean difference (for all inclines, including level ground) for IC was −2 ms (CI [−16; 12]) and for FO it was 35 ms (no confidence interval reported). Both studies reported the overall results considering both ramp up and ramp down walking (and level ground for Sabatini *et al*) as one condition, hence a direct comparison with the results in this study is not possible.

The results of the present investigation show differences in detection for the three terrains. They may be related to the changes in the kinematics of the knee for up and down incline walking with respect to level walking. In particular, at IC differences have been found in the angle of the knee (increased flexion for uphill walking) between upslope and level walking [29,30], whereas no changes have been reported between downhill and level walking [29,39]. In the present study, there is more discrepancy in IC detection between the level walking condition (−8 ms) and the uphill (incline up) condition (−21 ms), than between the level walking and downhill (incline down) (−9 ms) conditions. However, from terminal stance to FO the knee is more flexed during downhill than during level walking [29,31]. There is also a change in knee angle between the uphill and the level walking conditions (for uphill walking there is less flexion than for level ground), however the change with respect to level walking in knee angle is less pronounced than that for downhill walking [29]. Again the discrepancy in the present study for the mean difference between downhill (73 ms) and level walking (50 ms) is greater than the one between uphill (43 ms) and level walking.

It is noted that the reference system used for this study (FScan insoles) showed an absolute mean differences in event detection with respect to a gold standard (force platforms) of 22 ms for IC and 10 ms for FO. So the actual differences between gyroscope detection and the gold standard could be larger than the ones reported here (*i.e*., absolute mean differences could be 22 ms larger for IC and 10 ms larger for FO).

The algorithm proposed in this study is rule-based and evaluates sequentially each sample, which facilitates the conversion into an on-line algorithm, if the application requires it. However, the detection of FO requires the analysis of 15 samples ahead of the sample being evaluated. Consequently, if the algorithm were to be directly converted to an on-line process, the FO detection would have a delay of approximately 120 ms. If this delay was unacceptable for a particular application, further rules could be added (for example, the use of specific parameters for each subject such as a threshold on the gyroscope signal for FO detection).

### 3.2. Distribution of the Differences

**Figure 5.**
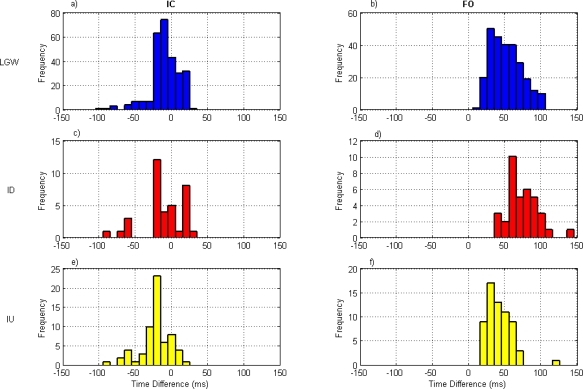
Frequency distribution of the time differences between the gyroscope and the reference method for IC and FO event detection for: level ground walking LGW (a) and (b); walking down the incline ID (c) and (d); and walking up the incline IU (e) and (f). The number of events considered were 273 IC and 266 FO for LGW, 36 IC and 36 FO for ID, and 63 IC and 63 FO for IU. Positive differences indicate that the gyroscope method detected the event earlier than the reference.

Figure 5 shows the histograms of the time differences in IC and FO detection for the different terrains. In order to avoid any bias in the histograms due to the different number of events detected for each subject, the maximum number of steps available for all subjects was considered. Therefore, in the case of level ground walking the first 39 IC events and 38 FO events were considered for each subject, giving a total of 273 IC and 266 FO. For walking down the incline, the first 6 IC and 6 FO events were considered for each subject; a total of 36 IC and 36 FO. Finally, for walking up the incline, the first 9 IC and 9 FO events were considered for each subject; a total of 63 IC and 63 FO.

The histograms show that there is a tendency for the IC differences to be negative whereas the differences for FO events are mainly positive and this remains true regardless of terrain. This tendency for the gyroscope to detect IC later than the reference and FO earlier than the reference is in agreement with other investigations [9,18,26,28].

### 3.3. Reliability

The CA method detected a total of 893 events (considering all IC and FO events) on level ground walking. Of those, the gyroscope missed or wrongly detected 4 events (all of them FO), so that 99.5% of events were detected successfully by the gyroscope on level ground walking. For walking down the incline, the reference method detected a total of 99 events of which the gyroscope missed one event (FO), so the number of correct events detected was 98.9%. In the case of walking up the incline, the total number of events detected was 147. The gyroscope missed one event (FO), so that the number of successfully detected events was 99.3%. Considering all terrains together, the gyroscope detection missed or wrongly detected a total of 6 events (all of them FO) from a total of 1135 events (99.5%).

Salarian *et al.* [27] reported on the sensitivity in detection of gait events using a gyroscope on the shank as 99.6% in healthy adults and 96.4% in patients with Parkinson’s disease. Smith *et al.* [38] reported the reliability of force sensing resistors as 94.5% in children with cerebral palsy. The algorithm used in this study detected correctly at least 98.9% of the events for each terrain and the errors in detection did not affect subsequent detection of events.

### 3.4. Final Considerations

The speed of walking was not standardized for different subjects in this study, which could affect the comparison between different terrains. Walking speed affects maximum knee flexion angles during stance [40,41], among other kinematic parameters. A treadmill could have been used as a way of overcoming the lack of speed standardization. However, treadmill walking also affects the kinematics of walking and spatio temporal parameters [42], especially the kinematics of the knee [43]. Researchers have found statistically significant differences in maximum flexion of the knee between treadmill and overground walking [44–46] and different position of the knee at initial contact [43]. Both variability of gait speed and the use of treadmill instead of overground walking have an effect of the kinematic of the knee. An outdoor path which included incline walking was regarded as a more natural environment. Future work should study the effect of walking speed on the gyroscope detection algorithm.

In addition, the differences in mean stride time for different terrains (1.07 ± 0.09 s for incline down, 1.08 ± 0.10 s for level ground and 1.10 ± 0.10 s for incline up) are similar to the differences between terrains obtained by Lay *et al.* [29] (1.17 ± 0.08 s for incline down at −15°, 1.20 ± 0.08 s for level ground and 1.23 ± 0.08 s for incline up at 15°), who studied gait kinematics and kinetics while walking on different inclines. So, since the variation in mean stride time between terrains in this study was 0.03 s, and the difference obtained by Lay *et al.* [29] were 0.06 s, it is considered that the modifications in knee kinematics presented in this study would be similar to those obtained by Lay *et al.*

The walking path used in this study included only one incline of 9°. Lay *et al.* [29] measured kinematic and kinetic variables on subjects walking on level ground (0° incline) and up and down inclines (8.5° and 21°). Of the fifteen variables measured, for the 8.5° incline, ten showed statistical difference for uphill walking and thirteen variables showed statistical difference for downhill walking when compared to level ground walking. For the 21° incline, seven showed statistical difference for uphill walking and nine variables showed statistical difference for downhill walking when compared to level walking.

Based on the results, Lay *et al.* [29] concluded that the transition between level walking strategy and incline walking strategy occurred below 8.5° (and suggested a value around 6°). Hence, it was considered that the subjects in this study used an “incline walking strategy” on the 9° incline, *i.e*., the incline used was considered sufficient to highlight any differences in accuracy and reliability of events detection due to differences in walking pattern.

A limitation of this study is that the CA method was evaluated on level ground. However, as it focuses on area loaded rather than actual location of pressure under the foot, no change in its accuracy is expected when used on inclines.

An interpretation of the accuracy of the results must be done in the context of the proposed application. This study is part of an overall project directed at the evaluation of gyroscopes as sensors for outdoor gait event detection and their use as part of equipment for functional electrical stimulation (FES) for foot drop and toe-walking correction. Foot switches activated by the contact (or lack of contact) of the foot with the floor are widely used as part of commercial stimulators. Foot switches often consist of one force sensitive resistor (FSR) placed under the heel or toe of the patient and connected through wires to the stimulator. However, it has been found that such a system contributes to difficulties operating FES systems and to the unreliability of the device [47], and different sensors have been evaluated to replace or augment the use of foot switches [1,15,48–50]. Although several studies have evaluated the accuracy of the sensors, there is very limited work that investigates the real effect of delays in detection on the results of stimulation. What is more, the effect of a delay in the stimulation timing depends on several variables, such as the condition of the neuromuscular system, the effect intended by the FES system (e.g., toe clearance, heel initial contact, or both), and the speed of walking. Ott *et al.* [51] reported on two cases of adult patients using FES for drop foot correction and compared the effects on stimulation of manual triggering against triggering with an FSR under the heel. For one of the patients, the hand switch was pressed, on average, 400 ± 200 ms after heel rise and released, on average, 100 ± 200 ms after initial contact and no significant difference was found in the basic gait parameters measured (stride time, stride length, speed and cadence). For the other patient the differences in triggering were larger (hand switch was pressed, on average, 1,100 ± 300 ms after heel rise, and released, on average 200 ± 100 ms after initial contact, and significant differences in the basic gait parameters were found (in fact, the subject walked faster, with increase stride length).

The differences between the gyroscope and the reference methods in this study are notably lower than the values reported by Ott *et al.*, suggesting that a shank mounted gyroscope is worthy of further investigation as a sensor in FES systems. In addition, from the results shown in Table 3, the gyroscope detection of FO was consistently earlier with respect to the reference. Future work could include in the detection algorithm a set delay in FO detection (of approximately 50 ms), which would reduce the differences between detections considerably.

## 4. Conclusions

Practical systems capable of accurate detection of gait events would be useful in many ambulatory applications. Gyroscopes placed at the shank have been proposed as part of ambulatory gait analysis and FES systems and have been evaluated on level ground. This study has focussed on the detection when walking outdoors on a path that included level ground and incline walking.

Although this is a preliminary study with a relatively small sample size, the results (absolute mean differences smaller than 25 ms for IC and better than 75 ms for FO, and reliability: over 98% for the three evaluated terrains) support the use of the gyroscope for detection of IC and FO walking on level ground and on inclines. Based on the results obtained, which are comparable in range to previous ones reported in the literature for a variety of event detection algorithms using different sensors, the authors believe that event detection through a gyroscope mounted on the shank is worthy of further study. However, future work should include evaluation of the sensor on paths that include stairs, turns, stops, walking at different speeds, and with subjects presenting with pathological gait.

## Figures and Tables

**Table 1.** Absolute mean difference ± one standard deviation (ms) in detection of Initial Contact (IC) using raw data and data low pass filtered from an unimpaired subject and one subject with CP. The cut off frequencies used were 5, 10, 12, 15, 20, 25, 30, 35 and 40 Hz. n = 34 IC events for unimpaired subject and 15 for subject with CP.

**IC**	**5 Hz**	**10 Hz**	**12 Hz**	**15 Hz**	**20 Hz**	**25 Hz**	**30 Hz**	**35 Hz**	**40 Hz**
Unimpaired	60.3 ± 34.3	26.5 ± 25.0	15.6 ± 20.0	12.0 ± 17.7	5.6 ± 7.0	4.7 ± 6.1	2.9 ± 4.6	2.3 ± 4.3	0.6 ± 2.4
CP	11.3 ± 5.2	3.3 ± 4.9	2.5 ± 4.6	3.3 ± 0.6	3.3 ± 0.6	3.3 ± 0.6	2.7 ± 0.6	4.0 ± 0.9	0 ± 0

**Table 2.** Absolute mean difference ± one standard deviation (ms) in detection of the Foot Off (FO) using raw data and data low pass filtered from an unimpaired subject and one subject with CP. The cut off frequencies used were 5, 10, 12, 15, 20, 25, 30, 35 and 40 Hz. n = 32 FO events for unimpaired subject and 13 for subject with CP.

**FO**	**5 Hz**	**10 Hz**	**12 Hz**	**15 Hz**	**20 Hz**	**25 Hz**	**30 Hz**	**35 Hz**	**40 Hz**
Unimpaired	10.9 ± 8.2	5.6 ± 6.2	4.1 ± 4.9	3.7 ± 5.5	2.5 ± 4.4	2.2 ± 4.2	1.8 ± 4.0	1.8 ± 4.7	2.2 ± 6.6
CP	4.6 ± 6.6	3.8 ± 5.0	3.8 ± 5.0	3.8 ± 5.0	3.1 ± 0.5	2.3 ± 4.3	1.5 ± 3.7	2.3 ± 4.4	2.3 ± 6.0

**Table 3.** Absolute mean difference (AMD) ± one standard deviation, Mean difference (MD) ± one standard deviation and 95% Confidence Interval (CI), all expressed in milliseconds, in the detection of Initial Contact (IC) and Foot Off (FO) between the gyroscope and the reference system. Positive MD indicates that the gyroscope method detected the event earlier than reference. n = 7 for level ground and walking up the incline, and n = 6 for walking down the incline.

	**Level Ground Walking**			**Incline Down Walking**			**Incline Up Walking**		
	AMD	MD	CI	AMD	MD	CI	AMD	MD	CI
IC	15 ± 6	−8 ± 9	[−16; 1]	20 ± 11	−9 ± 20	[−29;12]	24 ± 12	−21 ± 15	[−35; −8]
FO	50 ± 14	50 ± 14	[37; 63]	73 ± 12	73 ± 12	[60; 85]	43 ± 10	43 ± 10	[34; 52]
